# Copying You Copying Me: Interpersonal Motor Co-Ordination Influences Automatic Imitation

**DOI:** 10.1371/journal.pone.0084820

**Published:** 2013-12-31

**Authors:** Daniel Joel Shaw, Kristína Czekóová, Jakub Chromec, Radek Mareček, Milan Brázdil

**Affiliations:** 1 Behavioral and Social Neuroscience Research Group, CEITEC – Central European Institute of Technology, Masaryk University, Brno, Czech Republic; 2 First Department of Neurology, Faculty of Medicine, and St. Anne’s University Hospital, Masaryk University, Brno, Czech Republic; Royal Holloway, University of London, United Kingdom

## Abstract

Moving in a co-ordinated fashion with another individual changes our behaviour towards them; we tend to like them more, find them more attractive, and are more willing to co-operate with them. It is generally assumed that this effect on behaviour results from alterations in representations of self and others. Specifically, through neurophysiological perception-action matching mechanisms, interpersonal motor co-ordination (IMC) is believed to forge a neural coupling between actor and observer, which serves to blur boundaries in conceptual self-other representations and causes positive views of the self to be projected onto others. An investigation into this potential neural mechanism is lacking, however. Moreover, the specific components of IMC that might influence this mechanism have not yet been specified. In the present study we exploited a robust behavioural phenomenon – automatic imitation – to assess the degree to which IMC influences neural action observation-execution matching mechanisms. This revealed that automatic imitation is reduced when the actions of another individual are perceived to be synchronised in time, but are spatially incongruent, with our own. We interpret our findings as evidence that IMC does indeed exert an effect on neural perception-action matching mechanisms, but this serves to promote better self-other *distinction*. Our findings demonstrate that further investigation is required to understand the complex relationship between neural perception-action coupling, conceptual self-other representations, and social behaviour.

## Introduction

Humans have a tendency to co-ordinate their movements and behaviours with those of their interaction partners non-consciously. We either co-ordinate our own movements in time (synchrony), or we adopt the actions of those with whom we are interacting (mimicry; for reviews see [1] [2] [3]). There is a general consensus that both types of behaviour – herein referred to collectively as interpersonal motor co-ordination (IMC) – serve an important social function; namely, to promote social cohesion and affiliation [1]. Consistent with this perspective, performing synchronously with another individual, either consciously or unintentionally, engenders co-operative, altruistic, and affiliative behaviour towards them [4] [5] [6] [7]. Likewise, whether spontaneous or intentional, mimicry is shown to increase positive attitudes between mimicker and mimickee (for reviews see [2] [8]), and positive outcomes from social interactions (e.g. negotiations [9] [10]). These studies demonstrate that both forms of IMC are capable of modifying subsequent social behaviour. The task now is to elucidate the mechanisms underlying this phenomenon, and this was the aim of the current investigation.

It has long been postulated that one’s own and others’ actions are coded in a common representational space [11] [3]. Recent neurophysiological research supports this notion by revealing extensive spatial overlap in neural representations for self- and other-action; observing another individual performing an action engages our own neural motor circuits involved in executing that same action [12] [13] [14]. It is proposed that such “resonance” of neural motor systems during action observation activates corresponding intentional representations, permitting us to infer the actor’s goals and intentions [15], and, by extension, their mental and emotional states [16] [17]. Inferring that others are acting in way that conforms to our own intentions should promote a sense of affiliation. In this light, when our interaction partners perform actions that are synchronous or co-ordinated with our own, this should lead to the impression of shared mental and emotional states, and feelings of closeness and similarity [18]. In other words, during IMC, the activation of overlapping neural representations for self- and other-action may serve to blur boundaries in higher-level conceptual self-other representations, providing a mechanism through which positive views of the self are projected onto others [19] [20].

In line with this proposal, synchronised movements appear to blur cognitive self-other representations [21] and foster judgments of similarity and entitativity [22] [23] [24]. Furthermore, a similar effect is observed following multi-sensory stimulation; when we observe another individual exposed to tactile stimulation that is synchronized to that which we ourselves are experiencing, our bodily self-representation appears to extend and incorporate the other. In addition to this “enfacement” effect, we are more likely to perceive the other as similar to ourselves and experience greater closeness towards them [25] [26] [27]. As such, these studies demonstrate that perceiving synchrony between our own and another’s body influences both self-other overlap in bodily representations and social processing. Importantly, observing another individual being touched engages our own neural somatosensory circuits [28] [29] [30], suggesting that spatially overlapping neural self-other sensory representations underlie this effect. Taken together, such research suggests that IMC might influence behaviour by enhancing “self-other equivalence” in neural action representations [4]. To our knowledge, however, no investigation has explored this directly.

A large corpus of studies have demonstrated that one’s own actions are influenced greatly when we observe simultaneously those of another; we are quicker and more accurate performing actions that are congruent with those observed, while observing incongruent actions interferes greatly with our own action execution (for a comprehensive review see [31]). Importantly, the influence of others’ actions on our own occurs even when it is detrimental to the task at hand. It is considered, therefore, to reflect an automatic tendency to imitate the actions of others. As such, *automatic imitation (AI)* is considered an experimental manifestation of non-conscious mimicry [2] [8]. Furthermore, neuroscientific studies reveal that this influence of another’s actions on our own is driven directly by neural action observation-execution matching mechanisms [32] [33] [34]. Automatic imitation, then, also provides a method to investigate behaviorally if IMC alters social behaviour through its influence on these neural perception-action coupling mechanisms [31]. Recent studies that report an enhancement of AI following priming with pro-social (e.g. group, team, friend) relative to non-social word stimuli [35] [36] [37] validate such an approach (see [2]).

We utilized AI as a means to investigate behaviourally whether IMC exerts an effect on neural perception-action coupling mechanisms, and how this relates to subsequent social behaviour. Using electromyography, we examined whether observing another’s actions that match those we ourselves are executing simultaneously alters subsequent AI. Furthermore, we instructed individuals to perform their actions while observing an actor performing the same actions synchronously or asynchronously, or a different action synchronized temporally. By phase-shifting the observed actions relative to those executed, we were able isolate the temporal component of IMC; and by presenting different movements performed synchronously with the observers’, we manipulated separately the spatial characteristics of IMC. This permitted us to explore whether AI is influenced more by the spatial or temporal correspondence between our own actions and those of another (mimicry and synchrony, respectively). Finally, given that subjective perceptions of synchrony are reported to be more accurate than objective measures at predicting the subsequent effects on behaviour [5], we explored the influence of IMC on AI according to subjective reports.

Since prosocial behaviour is increased even when mimickers and synchronous partners are absent or inferred [5] [38], the actor’s actions were presented by video. This allowed us to manipulate IMC whilst controlling for other potentially confounding social factors (e.g. familiarity, interaction). Furthermore, subjects were given no information concerning the actor’s intentions, and they were instructed explicitly to execute their movements in time with an auditory rhythmic stimulus rather than the actor’s actions. By removing any instruction or intention to synchronise, we were able to investigate IMC as it occurs naturally. The present study, then, explored the effect of IMC on one individual’s neural action observation-execution mechanisms by manipulating the degree to which their actions corresponded to those of another individual in space and/or time.

## Materials and Methods

### Sample

The sample comprised 72 students (21 Males; mean age = 22.3 yrs, range = 19–36) recruited from various faculties of Masaryk University, Czech Republic. All participants were right-handed, reporting normal or corrected-to-normal vision and no neurological or psychiatric disorders. Written informed consent was obtained from every participant prior to the study, and the procedure was approved by the Ethics Review Board of St. Anne’s Hospital, Brno.

### Procedure

The experimental procedure comprised two stages: An initial period of interpersonal motor co-ordination (IMC) was followed immediately by a measurement of automatic imitation (AI). Throughout both stages, participants remained seated in a comfortable chair positioned 1 metre from a 24″ computer monitor located on a table in front of them. Before the first stage commenced, electrodes for electromyographic recordings were secured in place and the participant underwent 10 practice trials of the AI procedure (see below). Familiarising the participants with this latter aspect of the procedure at this early stage minimised errors on the task, and the gap between the two procedural phases.

### 1. Interpersonal Motor Co-ordination

In each of eight blocks, participants were instructed to perform one of two movements in time with an audible rhythm recording. In four of the blocks they were asked to tap the table in front of them with the index finger of their right hand; in the other half of the blocks, they were required to move from side to side their right hand in a waving action, with their arm positioned vertically and elbow resting on the chair arm rest, palm facing forwards. In both finger-tapping and hand-waving blocks, participants’ left hand rested in their lap. To ensure participants engaged fully in the task and to encourage perceptions of synchronicity, the audible recording presented during each block comprised one of two rhythms. Both rhythms lasted ∼60 secs and consisted of 12 repetitions of a 5-beat cycle, but differed slightly in their metric pattern.

Whilst performing the actions, participants observed the video-taped actions of an unfamiliar female actor on the monitor. Stimuli subtended a visual angle of 19.9×15.9°. In two conditions the video presented the actor performing the same hand-waving or finger-tapping actions as the participant, but with varying degrees of synchronicity. In the *Synch* condition, the actions were performed to the same audible rhythm aligned precisely to that followed by the participant; that is, stimuli were presented in-phase (0° phase shift). In the *Asynch* condition, the actor’s actions followed the same rhythm but phase-shifted; specifically, since only in-phase and anti-phase interpersonal synchrony is capable of modifying subsequent behaviour [22] [39], asynchronous stimuli were phase-shifted by ∼60–80°. Importantly, the degree of phase varied between videos but remained constant throughout each video. This way the actions of the actor and participant did not fall into synchrony with one another at any time during stimuli comprising the Asynch condition. In the *Temp* condition, the actor performed the opposite action to that of the participant (i.e. hand-waving actions while the participant executed finger-tapping, and vice versa), but to the same rhythm aligned precisely to that followed by the participant – i.e. in-phase. In other words, during the Temp condition participants observed different actions to their own but performed in temporal synchrony. Finally, in the *Control* condition the actor remained still throughout; her arm remained motionless in the vertical position during the hand-waving blocks, and her hand rested motionless on the table in front of her during the finger-tapping blocks. At the end of each block participants were also asked to rate on a 5-point scale the degree to which they felt the actor’s actions were synchronised “in time” with their own (1 = *completely unsynchronised*, 2 = *partly unsynchronised*, 3 = *partly synchronised*, 4 = *mostly synchronised*, 5 = *perfectly synchronised*).

**Figure 1:**
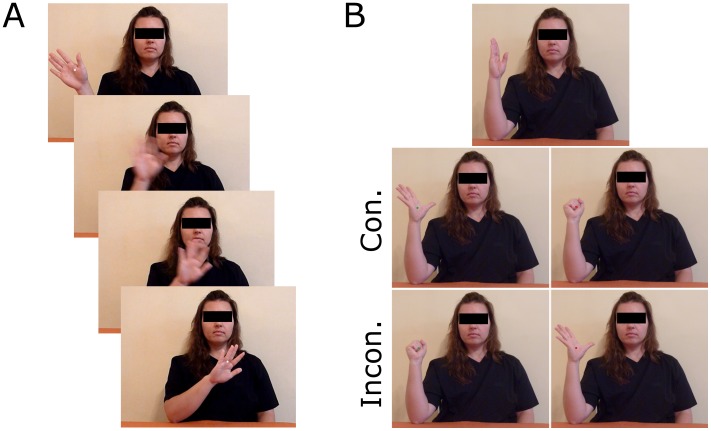
Visual stimuli. (*A*) Snapshots of the stimuli presented during the four hand-waving blocks of the IMC phase. Two instances of dots are presented (top and bottom frames) to illustrate that the dot-counting task demanded attention towards the actor’s actions. (*B*) Stimuli comprising the AI phase. Each trial began with the warning stimulus (*top*), after which the imperative stimulus (coloured dot) was presented, superimposed over congruent (*middle*) or incongruent (*bottom*) actions. NB: The actor has provided written informed consent, as outlined in the PLOS consent form, for the publication of this image. Although her identity is concealed in this figure, her eyes were visible throughout all experimental stimuli.

To ensure equal attention was paid to all types of stimuli, and to encourage focus on the actor’s movements, 2–4 small white dots were presented briefly (100 msec) in the vicinity of the actor’s moving hand or finger. Participants were asked to count the number of dots, and instructed to report the number of dots at the end of each block. Snapshots of the visual stimuli are presented in Figure 1.

### 2. Automatic Imitation

To measure automatic imitation we employed a typical stimulus-response compatibility procedure, in which the stimulus set included photographic images of the actions comprising the response set [40] [41]. With their right arm now positioned beside them, resting horizontally on the arm rest, participants were instructed to execute as fast as possible hand-opening or -closing actions in response to coloured dots. Participants were instructed to begin each trial with their hand in a ‘start’ position, in which the fingers and thumb were extended parallel to one another, forward facing. From this position, they were told to execute a hand-opening or -closing response as soon as they observed a green or red dot on the screen, respectively. The former action involved the extension and splaying of the fingers away from the palm, while the latter was achieved by rolling the fingers into a fist (see Figure 1).

The beginning of every trial was signalled by the presentation of the same female actor seated at the same table as before, her hand in the start position but her arm perpendicular to the table. This ‘warning’ stimulus was presented for 800, 1600, or 2400 msec, after which the actor’s hand changed to the end-point of the same hand-opening or –closing movement comprising the response set. The coloured dots were presented superimposed over the actor’s hand at the exact time it changed from the start position to the movement end-point. This meant that imperative stimuli (coloured dots) were presented alongside the task-irrelevant actions of the actor. On compatible trials, the actor performed the same action signalled by the imperative stimuli, while incompatible trials presented an incongruent action.

The shift from start position to movement end-point produced apparent motion. These static stimuli were selected over videos because (1) it allowed us to eliminate the possibility of unavoidable cues as to the upcoming movement (e.g. changes in the actor’s posture or facial expression), and (2) automatic imitation has been demonstrated repeatedly with static stimuli [31]. The action end-point and imperative stimuli were presented for 1500 msec. With the actor’s arm positioned vertically, their hand-opening and -closing actions involved moving their fingers along the horizontal plane, orthogonal to that of the participants’. This eliminated the possibility of spatial-compatible effects [42] [43]. Participants underwent two blocks of 60 trials, 30 congruent and 30 incongruent. Errors (i.e. hand-opening or -closing on a hand-closing or -opening trial, respectively) were recorded manually.

### Prosociality

As measures of prosociality, at the end of the procedure we asked our participants to rate the actor on two dimensions, both measured on a five-point Likert scale. First they were asked how likeable they considered the actor to be “in real life” (1 = *“Extremely unlikeable, we would never become friends”*; 5 = *“Extremely likeable, we could become close friends very quickly”*). This is analogous to the measure of likeability used elsewhere [4] [44]. Then we obtained a measure of willingness to co-operate. We assumed that our attitudes towards another individual drive our judgements concerning the efficacy of co-operating with them, and, therefore, our willingness to co-operate with them. On the basis of this assumption, participants were given the following (written) hypothetical scenario: *“Imagine that you and the same actor are asked to build a complicated 3-dimensional structure from LEGO*®. *You must give verbal instructions to the actor, who must follow your instructions blindfolded. How successful do you think this co-operation would be?*” (1 = *“Completely unsuccessful, we would never achieve the task”*; 5 = *“Completely successful, we would achieve the task in minimal time”*).

### Electromyography

Using AG/AgCl surface electrodes positioned in a belly-tendon montage, we recorded the onset of hand-opening and -closing actions by recording the electromyograph (EMG) from the first dorsal interosseus muscle of the right hand. The EMG was amplified and sampled at 1024 Hz, with no filtering applied. The signal was segmented into 2000 msec epochs, comprising a 500 msec baseline period and encompassing the signal recorded during the following 1500 msec post-imperative period. Within each epoch, reaction time was defined as the onset of a hand movement. This was measured by moving in 1 msec increments a 20 msec sliding window through the post-imperative period; movement onset was defined as the start of the first window in which the standard deviation for that window, and that of the next window, exceeded 2.5 times that of the baseline. An individual who was blind to the trial type inspected each epoch visually, rejecting any trial in which movement onset was not identified accurately.

## Results

**Figure 2:**
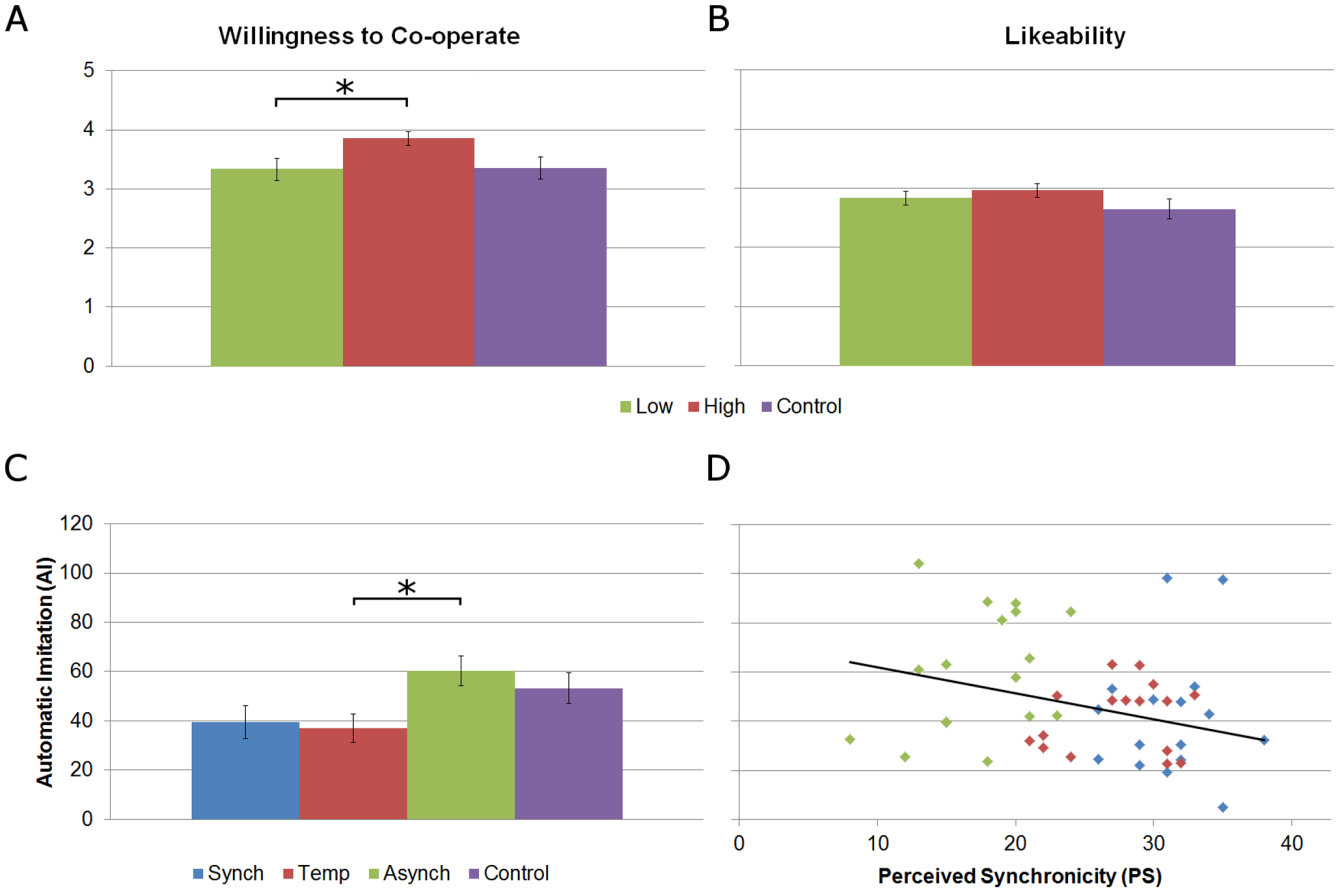
Primary results. (*A* and *B*) Results of Mann-Whitney tests, revealing a greater willingness to co-operate and enhanced likeability ratings in participants who provided higher ratings of PS; * = p<.05. (*C*) Results of one-way ANOVA, illustrating reduced AI in the Temp compared with the Asynch condition. Error bars present standard error; * = p<.05. (*D*) Results of the regression analysis, illustrating that AI decreases as a function of increasing subjective ratings of synchronicity. Regression line: *AI* = −1.05 *PS*+74.49.

Due to a poor electromyogram resulting from electrode displacement during the performance of the hand actions, we were forced to omit from our analyses the recording of one participant. In order to maximise the number of trials comprising each trial type (i.e. Incongruent and Congruent) we also excluded from our analyses three subjects who made more than 10 errors. In the sections that follow, we report the results of parametric statistical analyses unless the assumptions of normality and/or homogeneity of variance were violated. Measures are presented as means (± standard deviation). Figure 2 illustrates the primary findings of our analyses.

To evaluate the effectiveness of our manipulation we performed a one-way ANOVA on ratings of Perceived Synchronicity (PS) summed over all eight blocks (max. = 40), with Condition as a between-subject factor. Since PS ratings were not acquired for the Control condition, we examined only the Synch, Asynch and Temp conditions. This revealed a main effect of Condition (F_(2,48)_ = 55.00, p<.001; η^2^ = .70), with post-hoc tests (Tukey’s HSD) revealing significant differences between the Synch (33.06 [±3.36]) and Asynch condition (19.35 [±4.31]; p<.001), and the Asynch and Temp (29.94 [±4.32]) conditions (p<.001). Surprisingly, a Kruskal–Wallis test revealed no Condition effect on ratings of likeability (H_(3)_ = 2.88, p = .42; Synch = 2.94 [±.66], Asynch = 2.82 [±.53], Temp = 2.94 [±.54], and Control = 2.65 [±.70]) or willingness to co-operate (H_(3)_ = 3.16, p = .37; Synch = 3.71 [±.67], Asynch = 3.35 [±.86], Temp = 3.76 [±.83], and Control = 2.75 [±.77]). When we performed a median split on the basis of PS ratings, however, a Bonferroni-corrected Mann-Whitney test revealed significantly higher willingness to co-operate in individuals with higher (3.85 [±.60]) relative to lower PS ratings (3.33 [±.92]; U = 229, p_corr_ = .046). Furthermore, although not significant, we observed greater likeability ratings for participants with high PS ratings (2.96 [±.59]) compared with those in the Control condition (2.65 [±.70]; U = 168, p_corr_ = .094).

To assess whether equivalent attention was paid to all types of stimuli, we examined performance on the dot-counting task by adding the reported number of dots counted across all eight blocks. This revealed a very small number of errors in all conditions; of the 27 dots presented across all blocks, the mean number of totals errors in the Synch, Asynch, Temp and Control condition was only 1.71 (±1.45), 2.29 (±1.16),.88 (±1.05), and 1.53 (±.94), respectively. Applying a non-parametric Kruskal-Wallis test to the number of dot-counting errors, however, with Condition a between-subject factor, revealed a significant effect (H_(3)_ = 13.15, p = .004). Bonferroni-corrected follow-up tests revealed that this was driven by the difference between the Temp condition and the Asynch (p_corr_ = .007) and Control (p_corr_ = .030) conditions.

We turn now to our assessment of automatic imitation (AI). As expected, a two-way ANOVA, with Condition a between-subject factor and trial type (Trial) a within-subject factor, confirmed that more errors were made on incongruent (4.07 [±2.07]) compared with congruent trials (1.26 [±1.36]; F_(1,64)_ = 79.74, p<.001). The same test confirmed that the number of errors were equivalent across all conditions, however – there was no effect of Condition (F_(3,64)_ = 1.29, p = .29), and no Condition-by-Trial interaction (F_(3,64)_ = .59, p = .62). As a measure of AI, we subtracted reaction times on congruent trials from those on incongruent trials. As such, larger values represent greater AI. A one-way ANOVA observed a significant main effect of Condition on AI (F_(3,64)_ = 3.23, p = .028, η^2^ = .13), with post-hoc tests (Tukey’s HSD) revealing a slight decrease in the Synch compared with the Asynch condition (39.43 [±27.36] vs. 60.22 [±24.94] msec, respectively; p = .09), and a significant decrease in the Temp (37.07 [±23.88] msec) relative to the Asynch condition (p = .048; Control = 53.25 [±25.38] msec). Interestingly, this decrease in AI following the Synch and Temp conditions relative to the Asynch condition was present in the first testing block (F_(3,64)_ = 3.26, p = .027, η^2^ = .13; Temp [38.60±24.40] *vs.* Asynch [64.75±29.07], p = .029; Synch [42.62±29.95] *vs.* Asynch, p = .084; Control = 52.86 [±22.68]) but not in the second (F_(3,64)_ = 1.90, p = .138; Synch = 37.23 [±30.58], Asynch = 55.08 [±27.37], Temp = 35.42 [±27.72], Control = 52.32 [±34.83]). A 2-way ANOVA, however, with Condition a between-subject factor and Block a within-subject factor, revealed no main effect of Block (F_(1,64)_ = 2.11, p = .15), nor a Block-by-Condition interaction (F_(1,64)_ = 0.36, p = .78). For this reason, the following analyses were performed on AI collapsed across both testing blocks.

Spearman correlations revealed no relationships between AI and likeability (r_s_ = −.12, p = .35) or willingness to co-operate (r_s_ = −.01, p = .86). Likewise, a median split of AI values revealed no significant difference between participants with low relative to high AI in either willingness to co-operate (3.62 *vs.* 3.47, respectively; U = 524.50, p = .24) or likeability (2.82 *vs.* 2.85, respectively; U = 568, p = .49).

Next, we examined whether the modulation of AI was simply a result of the attention (i.e. dot-counting) effect. Specifically, we performed ANCOVA to see whether the reduction in AI between the Temp and Asynch conditions remained when the number of dot-counting errors was treated as a covariate. A significant Condition effect on AI (F_(3,63)_ = 6.37, p<.001, η^2^ = .23) confirmed that the modulation of AI was independent of dot-counting performance. The corrected means (± SE) for the Synch, Asynch, Temp and Control conditions over both testing blocks were 36.10 (±5.82), 64.21 (±5.85), 32.49 (±5.89) and 57.21 (±5.89) msec, respectively.

Given the apparent importance of the temporal aspect of IMC, we decided to examine the relationship between AI and PS for individuals comprising the three conditions in which PS ratings were obtained (Temp, Synch and Asynch). This revealed a weak but significant negative association (ß = −1.05, t = −2.01, p = .05, R^2^ = .08), indicating that AI decreased with increasing subjective ratings of synchrony. As a final step we investigated whether or not PS served as a mediator of the Condition effect on AI. To assess this we implemented a mediation analysis capable of estimating the direct, indirect, and total effects of multicategorical variables on continuous outcome measures (MEDIATE SPSS Macro) [45]. Through indicator (“dummy”) coding, this tool permitted us to capture the three relevant levels of Condition by modelling each as a predictor variable. In light of the above ANOVA results, the Asynch condition served as a reference against which the other levels were compared. In doing so, we were able to compare simultaneously the relative indirect effects of PS on AI when observing actions that are synchronous with our own either spatially *and* temporally (Synch) or temporally only (Temp), relative to asynchronous actions (Asynch). The statistical significance of indirect effects via the mediator variable is assessed with percentile confidence intervals from a bootstrapping procedure. In the current implementation, since both 95% intervals contained zero, the indirect effect cannot be considered to differ significantly from zero and the null hypothesis cannot be rejected: although subjective perceptions of synchrony are related negatively to AI, they do not mediate the Condition effect. These results are presented in Table 1.

**Table 1:** Estimates of mediatory effect of perceived synchronicity.

Condition*	Effect	SE	CI*_lower_*	CI*_upper_*
**Synch**	4.24	11.44	−15.08	22.24
**Temp**	5.49	14.71	−18.89	29.42

Estimate values after 1000 bootstrapped resamples.

= The Asynch condition served as the reference group;

SE = standard error; CI*_lower_* and CI*_upper_* = 95% lower- and upper-level confidence interval, respectively.

## Discussion

In this study we set out to investigate whether interpersonal motor co-ordination (IMC) influences neural perception-action coupling mechanisms. To do so, we examined whether IMC influences automatic imitation (AI) – a behavioural phenomenon that has been shown to result from the resonance of neural motor circuits during action observation [31] [32] [33] [34]. Our data reveal three interesting findings. Firstly, consistent with many previous studies [4] [7] [23] our operationalisation of IMC was successful in enhancing positive attitudes towards the actor; specifically, we observed a greater willingness to cooperate with the actor when their actions were perceived to be more synchronised in time with the participants’. Secondly, AI was reduced following a short period of observing the actor’s actions when they were synchronous with the observers’, relative to those that were asynchronous. Third, the relative increase in willingness to co-operate and reduction of AI was most evident when the observed actions were synchronised temporally with those executed, but differed in their spatial kinematics (i.e. the Temp condition). Furthermore, AI decreased with increasing subjective judgements of temporal synchronicity. This attenuation of AI was unrelated to either of our measures of pro-sociality, however. We interpret our findings as evidence that IMC does indeed influence neural perception-action coupling mechanisms, but this manifests as enhanced self-other *distinction* rather than overlap. Moreover, the way in which such modification of neural self-other representations influences social behaviour is less straightforward than generally assumed. Before we can begin to consider possible mechanisms driving this effect, we must first address some potential methodological explanations.

Although very small in magnitude, we observed a greater accuracy on the dot-counting task in the Temp relative to the Asynch and Control conditions. At first glance it might appear as though our modulation of AI is an artefact of differential attention paid to the stimuli defining these conditions. This would be consistent with studies that report preferential attention for interaction partners that move synchronously relative to those that move asynchronously with ourselves [46]. Importantly, however, our modulation of AI remained significant after controlling for this attention effect. Moreover, given that this difference in dot-counting would reflect increased attention to the stimuli defining the Temp condition, we consider this explanation insufficient; if greater attention was paid to the synchronous compared with the asynchronous stimuli, why should they influence subsequent behaviour to a lesser degree?

Instead, our results suggest that this dot-counting difference reflects the difficulty of the task under these two conditions. Precisely because of AI, counting the dots (and therefore attending to the actor’s actions) in the Asynch condition interfered with movement timing. In contrast, participants in the Temp condition could use the actions of the actor to assist them on both tasks – to predict where the next dot would occur and to keep in time with the auditory stimulus. The question still remains, however, why performance on the dot-counting task during the Asynch condition did not differ also from that measured during the Synch condition. Our interpretation would suggest that others’ actions interfere less with our own when they are synchronised temporally but differ in their spatial kinematics. This makes intuitive sense: Joint-action tasks often require us to perform actions that are distinct from those of our interaction partner, but co-ordinated in time. While temporal synchronicity would facilitate this by allowing us to predict our partner’s subsequent actions [3] [15], the tendency to imitate the spatial aspects of their actions would be detrimental.

A more feasible methodological explanation for our pattern of results relates to the findings of [6]. These authors discovered that a period of acting synchronously with another individual enhanced perceptual sensitivity, as measured by improved performance on a visuo-motor task. Perhaps the temporal co-ordination between the actor’s and subjects’ actions comprising the Temp condition served to hone our participants’ visuo-motor skills. This would allow them to respond more accurately to the imperative stimuli and ignore more easily the task-irrelevant, conflicting actions of the actor, thereby *decreasing* AI. This explanation can be tested empirically in a simple extension of our study. [42] [43] [47] observed AI even when subjects were instructed to produce a single, pre-defined movement in response to simple imperative stimuli. In these studies, subjects were required to make a single response in each block – *either* a hand-opening *or* -closing action – as soon as the imperative stimulus appeared. The imperative stimulus in these studies was a hand performing either the same (congruent trials) or opposite movement (incongruent trials) as the predefined action. In other words, these authors report AI even when the demands of stimulus-response mapping are minimised. If the shift in AI we have revealed following temporal synchrony is driven by enhanced perceptual sensitivity, we would expect this effect to be less evident in such a measure of AI.

Let us now speculate on some potential neurophysiological mechanisms that might underlie our manipulation of AI. One particular brain network is assumed frequently to be a likely candidate for producing “self-other equivalence” [4]. Within the mirror neuron system (MNS), observing the actions of others engages the observer’s own neural motor circuits in a corresponding fashion [12] [13] [14]. Within this brain system, then, others’ actions are coded and represented in the same way as our own, creating a neural coupling between actor and observer [4]. For this reason primarily, the MNS is assumed generally to serve as the primary neurophysiological mechanism through which IMC influences subsequent social behaviour [2] [3] [4] [18]. In support of this, imitative behaviour engages the MNS maximally [48]. Furthermore, temporary disruption to the MNS reduces AI [32], suggesting that AI is a product of the automatic resonance of motor circuits during action observation. It follows that if IMC influences social behaviour via MNS functions, IMC should also *enhance* AI. The findings of our study do not support this prediction, however, pointing to the involvement of alternative neurophysiological mechanisms.

One alternative mechanism is suggested by studies that have compared brain function during congruent and incongruent trials on the same stimulus-response compatibility procedure employed here. These studies have revealed the areas of the brain involved in the *control* of automatic imitation; in particular, inhibiting the tendency to imitate engages the medial prefrontal cortex and temporo-parietal junction [49] [50] [51] [52]. Patients with damage to these structures demonstrate stronger imitative response tendencies than those with damage to other brain regions [53]. Interestingly, these same brain systems are implicated heavily in high-level social cognitive processes, such as mentalising, perspective taking, and self-referential processing [54] [55] [56] [57]. As such, the neural mechanisms involved in the control of imitative response tendencies overlap with those underlying sophisticated self-other representations involved in meta-cognitive processes [51] [55]. In line with this, a study by [58] suggests that individuals with autism – a developmental disorder characterised in part by deficits of mentalising and perspective taking, and functional alterations in corresponding brain structures – may exhibit larger AI than healthy controls. It seems feasible to conclude that greater imitative tendencies in these individuals are the result of dysfunction in neural systems involved in high-level self-other representational systems.

Continuing with this notion of imitative control, our study demonstrates that observing actions co-ordinated temporally with our own inhibit subsequent AI more than those synchronised both in time and in space. We argue that the spatial discrepancy between the observed and executed actions in the Temp condition served to permit better self-other *distinction*. Although the timing of the actor’s movements in this condition assisted individuals in maintaining synchrony with the auditory rhythm, the spatially incongruent movements also trained participants to inhibit the tendency to imitate. In this sense, it is entirely conceivable that our Temp condition operated in an analogous manner to the imitation-inhibition training implemented by [59]; these authors report that training participants to overcome the automatic tendency to imitate others not only reduced AI, but also *improved* perspective-taking ability. This interesting effect is attributed to improvements in self-other distinction, allowing participants to resist the influence of others’ actions on their own motor planning on one hand, while on the other enabling them to distinguish between their own and others’ perspectives. The results of our study can be seen as an extension of this work to the domain of IMC, whereby the observation of spatially incongruous but temporally synchronised actions reduce AI and enhance positive social attitudes. The questions remains whether the same brain systems involved in imitative control also underlie the attenuating effect of IMC on AI observed in the present study, and how this influences social behaviours. This demands further neuroscientific investigation.

Moreover, to make the claim that our manipulation of AI reflects improved self-other distinction, it is necessary to examine whether such an effect corresponds to other measures of self-other representation. Studies have utilised the “enfacement” effect to explore the expansion of bodily self-representations [25] [26] [27], the “self-referencing” effect to demonstrate self-other merging in memory [24], and the Inclusion of Other in Self (IOS) scale [60] [61] to capture subjective feelings of self-other overlap [25]. Future investigations are needed to explore whether the alterations in a behavioural index of neural self-other representations following IMC that we have demonstrated are reflected also in these higher-level measures of self-other overlap.

Nevertheless, the findings of the present study offer an important contribution not only to research concerning IMC, but also to our understanding of self-representation. Participants in our Temp condition experienced temporally but not spatially correlated visuo-motor inputs. This condition, then, is analogous partly to the multi-sensory stimulation used by [62]. These authors report “enfacement”, enhanced perceptions of physical similarity, and greater feelings of closeness when they experienced unrelated but temporally synchronous visual and tactile stimulation. Such overlapping bodily representations may allow us to “feel” first-hand the sensory experiences of others (e.g. pain). Like this and other related studies [25] [26] [27], our findings suggest that self-representations are flexible rather than stable, and malleable to *temporally* synchronised multi-sensory input. In the action domain, however, temporal synchronicity appears to permit better self-other distinction rather than overlap. This, conceivably, is more adaptive for real-life co-operative interactions, whereby we must act in a manner that complements rather than mirrors the motor behaviour of our interaction partners.

Finally, although we observed a significant increase in willingness to co-operate in those reporting more relative to less perceive temporal synchrony between their own actions and those of the actor, there was no such modulation of likeability. Further, we revealed no relationship between AI and liking, despite employing a measure similar to that used elsewhere [4] [44]. We interpret this according to methodological differences between these studies and our own. First, to isolate the effects of spatial and temporal motor co-ordination we employed stimuli that prevented any social interaction between the actor and observer. Perhaps, however, it is through social interaction (e.g. eye-contact, sharing facial expressions, and exchanging utterances) that we express the sharing of an experience necessary to engender feelings of similarity and closeness. Importantly, the positive effects of IMC on social behaviour appear to be modulated by perceptions of similarity [21] [23]; perceptions of dissimilarity and other negative attitudes towards interaction partners reduces the potential for synchronicity [21], the frequency of mimicry [63] [64] [65] [66], and the potential for these variables to modify subsequent social behaviour [64] [65]. Secondly, subjects were aware that the stimuli were offline videos of the actor; although synchronised with their own movements, they were aware that the actor had performed her actions in the past. Such awareness might have limited the degree to which overlapping neural self-other action representations were capable of modulating subsequent social behaviour towards the actor. Although we can mentalise and empathise with real-life characters on the TV, we have a meta-cognitive awareness that their experiences have occurred in the past and in no way relate to those we ourselves are experiencing in the present. This may prevent any enhancement of feelings of closeness, similarity, or affiliation. To investigate this further, we suggest an extension of our study in which the actor is present and in contact with the observer during the IMC phase, and performs the task-irrelevant hand actions in real-time during AI assessment.
